# Geo-demographics of gunshot wound injuries in Miami-Dade county, 2002–2012

**DOI:** 10.1186/s12889-017-4086-1

**Published:** 2017-02-08

**Authors:** Laura Zebib, Justin Stoler, Tanya L. Zakrison

**Affiliations:** 1 Department of Epidemiology, Mailman School of Public Health, Columbia University, 722 W 168th Street, New York, NY 10032 USA; 2 Department of Geography, University of Miami, 1300 Campo Sano Avenue, 115H, Coral Gables, FL 33146 USA; 3 Department of Public Health Sciences, Miller School of Medicine, University of Miami, Miami, FL USA; 4 Department of Surgery, Ryder Trauma Center, Jackson Memorial Hospital, University of Miller School of Medicine, University of Miami, T247 1800 NW 10th Ave, Miami, FL 33136 USA

**Keywords:** Miami, Gunshot wound, Gun violence, Racial disparities, Spatial analysis

## Abstract

**Background:**

We evaluated correlates of gunshot wound (GSW) injuries in Miami-Dade County, Florida. Firearm-related injury has previously been linked to socio- and geo-demographic indicators such as occupation, income, neighborhood and race in other metropolitan areas, but remains understudied in Miami.

**Methods:**

We reviewed 4,547 cases from a Level I trauma center’s patient registry involving an intentional firearm-related injury occurring from 2002 to 2012. During this eleven-year study period, this trauma center was the only one in Miami-Dade County, and thus representative of countywide injuries.

**Results:**

The crude morbidity rate of GSW injury over the 11-year period was 15 per 100,000 persons with a crude mortality rate of 0.27 per 100,000 persons. The case fatality rate of injured patients was 15.4%. Both morbidity and mortality increased modestly over the 11-year study period. The total number of GSW patients rose annually during the study period and patients were disproportionately young, black males, though we observed higher severity of injury in white populations. Geo-demographic analysis revealed that both GSW incident locations and patient home addresses are spatially clustered in predominantly poor, black neighborhoods near downtown Miami, and that these patterns persisted throughout the study period. Using spatial regression, we observed that census tract-level GSW incidence rates (coded by home address) were associated with a census tract’s proportion of black residents (*P* < .001), single-parent households (*P* < .001), and median age (*P* < .001) (*R* ^2^ = .42).

**Conclusions:**

These findings represent the first representative geo-demographic analysis of GSW injuries in Miami-Dade County, and offer evidence to support urgent, targeted community engagement and prevention strategies to reduce local firearm violence.

## Background

In 2011, 478,400 fatal and nonfatal firearm violence incidents were reported within the United States, where firearm violence accounts for over 11,000 deaths annually [1, 2]. Several national studies and retrospective reviews have revealed clear racial disparities in both prevalence and outcomes for the victims of gunshot wounds (GSW), who are disproportionately adolescent, black males [3–7]. In fact, firearm violence is the leading cause of death for African-American men ages 15–34 [8]. Several studies have observed that black patients have a higher mortality rate and worse outcomes than white patients involved in interpersonal violence [9–12]. Class, as it overlaps with race, is also associated with firearm violence, especially in societies with increasing disparities between rich and poor neighborhoods [13]. National trends include overrepresentation of patients of lower socioeconomic status, and most violence occurring in neighborhoods with concentrated poverty and housing density [5, 14–17]. While studies in large cities have produced results similar to the national trends in terms of age, race, and geographic clustering, there has been little consensus of temporal trends in firearm-related injury in both pediatric and adult populations [3, 10, 18, 19]. However, it is unclear if these temporal increases or decreases in firearm-related injuries are consistent across all races and neighborhoods over the last decade [7, 20].

Geospatial analysis of firearm-related injuries provides additional evidence that gun violence is typically clustered in low-income urban centers with spatial decay into less urban areas [21]. The use of a GIS (geographic information system) to visualize and analyze firearm-injury as a geo-social issue provides greater insight into the determinants of violence and has shown that the majority of gun violence occurs in as little as 3% of the total metropolitan area [22]. Furthermore, sub-analysis of geocoded data has provided insight into social phenomena such as the “journey to violence” which describes how firearm-related crimes frequently involve mobility away from the home [23]. It has been shown that gun violence within urban areas typically occurs in streets, with victims often in neighborhoods outside of their own [23, 24]. This is important for identifying so-called violence “hot spots” that can be targeted for injury and violence prevention in different cities across the United States.

Miami-Dade County (MDC) has a long-standing history of firearm violence, with firearm related injury accounting for 11.6% of admissions at a Level I Trauma Center (unpublished data). Yet there have been virtually no studies of firearm violence within MDC over the past decade, with most previous literature reviewing crime data that typically lacks the victim’s home address. To our knowledge, no geospatial analysis of the victim profile of gun violence has ever been performed for MDC, despite being performed in other metropolitan areas. In contrast to other large cities, Miami provides a unique ethnic and racial makeup with a predominately Latin-American population. Such urban diversity has given rise to concentrated ethnic enclaves with well-defined geographical boundaries, including large Cuban communities in Little Havana and Hialeah, a Haitian enclave in Little Haiti, and large African-American communities in Carol City, Opa-Locka, Liberty City, and Overtown [7, 20]. Thus, race, ethnicity, and neighborhood are interlaced within MDC. These racial and ethnic enclaves reinforce complex economic and social structures that are built on ethnic pride and cultural unity. Many argue that this segmented assimilation into enclaves offers a number of positive benefits for residents including a stabilized economy and decline in drugs, crime, and violence [7, 25]. Others suggest that Miami’s social organization perpetuates intra-racial disagreements and social phenomenon, which increases violence within certain racial groups [20, 26]. To date, there are no data in the scientific literature about the temporal or geographic trends of firearm violence in Miami Dade County, which has a homicide rate that is 200% higher than the national average [27].

The history of black communities within MDC is essential to understanding the high rates of violence that have developed in these areas. During the development of Miami during the early 20^th^ century, black workers were recruited from the Bahamas, Mississippi, Alabama, and Georgia to clear mangroves and build new roads to cater to transient wealthy white residents, thus making black residents the original Miami locals [28]. During this time period, the “color line” was drawn to restrict black residents from expanding out of what is now known as Overtown. This segregation remained in Miami into the late 1960s, and protests by white residents against black mobility into white neighborhoods in the first half of the century led to increased population density within these predominantly black residential areas. Despite pervasive discrimination that prevented black families from moving into white neighborhoods, commerce within Overtown thrived as the heart of the local black community in Miami, with 389 businesses in 1950. However, the construction of I-95 and I-395 divided Overtown and ultimately led to the displacement of 12,000 people, only 5,000 of whom had the economic resources to relocate themselves [29]. Thus the population left behind was predominantly poor black families who lacked the resources to move out of the newly created dead space, and only 41 of 389 thriving businesses remained. Many individuals migrated to Liberty City where the majority of public housing projects from the 1930s were located, thus turning it into an urban ghetto and increasing the competition for affordable housing and jobs with new Cuban immigrants. Overtown now has the highest rate of poverty and some of the worst housing in South Florida, and Liberty City has remnants of concrete walls used to separate black and white communities, as well as the largest concentrations of black residents in MDC. This legacy, combined with local neighborhood characteristics, such as high housing density and poverty, has facilitated a culture of violence. In fact, these areas have garnered so much national attention for their high rates of gun violence that the City of Miami erected cautionary road signs in the 1990s to warn tourists from entering these areas. While MDC has followed the national trend of an overall reduction of gun violence, high rates of violence still persist in these low-income areas.

As little is known about firearm-related injury within MDC, this paper presents the socio-demographic and geospatial patterns of firearm violence within MDC over 11 years. This is the first step toward identifying communities that are most in need of an integrated violence reduction strategy. This paper reviews the profile of GSW victims treated from 2002 to 2012 by a major Level I Trauma Center, which, during this period, was the only trauma center in the county. We hypothesized that firearm violence, both fatal and non-fatal in MDC, is confined to narrowly-defined geospatial tracts with minimal change over time, thus providing an excellent opportunity for policy-level change for injury and violence prevention.

## Methods

### Data

We reviewed the trauma registry at Jackson Memorial Hospital’s Ryder Trauma Center in Miami, FL, for all patients treated for a GSW from January 1, 2002 to December 31^st^, 2012 using International Classification of Disease coding (ICD-9). This medical center was the only Level I Trauma Center within MDC during the study period, and 5,412 patients sustained a firearm-related injury. Each entry represents a separate firearm incident regardless of a patient’s previous trauma history. 865 patients whose GSW were self-inflicted, accidental or unintentional, originated from a BB gun (a very small caliber air or pellet gun), or who were transferred from or resided outside of MDC were excluded from the study. The remaining 4,547 patients were identified using the trauma registry as intentional, interpersonal firearm-related injury occurring within MDC. Of these 4,547 records, we were able to geocode 3,908 (86%) home addresses, and 2,788 (61%) incident addresses.

Data collected during treatment included date, age, sex, race, home address, incident address, occupation, admitting diagnosis, intensive care unit (ICU) length of stay, injury location, and mortality. Black patients include those of African-American and African-Caribbean descent. These data generally represent MDC’s GSW burden during the study period, but they do not include patients who did not seek medical attention.

### Analysis

Trauma scene and patient home location were used to calculate the distance between the injury location and patient’s home, thus enabling analysis of frequency of firearm violence within neighborhoods. Data from the 2010 US Census were used to provide descriptive socioeconomic measures at the census tract scale including race, age, average income, unemployment rate, housing density, number of single families, and total population. GSW incidence for the entire study period was defined as the number of incident cases in a census tract per 1,000 persons (using 2010 US Census counts). GSW incidence was calculated separately using both home and incident addresses. The crude mortality rate and case fatality rate were also separately calculated for each census tract using both the incident and home address.

All mapping and spatial analyses were performed using ArcGIS 10.3. The study area included only incidents and home addresses in Miami-Dade County. Census tracts with nominal populations on the fringe of the Florida Everglades and around Miami International Airport were excluded from analysis. The distance traveled from home address to incident address was calculated for each patient when both addresses were available. We analyzed temporal trends by computing descriptive spatial statistics such as the mean center and standard distances ellipse by year. Choropleth maps of morbidity, mortality, and case fatality rates at the census tract level were qualitatively compared with MDC data on demographics, employment rates, and housing density from the US Census Bureau. We used traditional spatial autocorrelation statistics, such as Moran’s *I* and local indicators of spatial autocorrelation (LISA), to assess MDC census tracts for spatial patterning of incidence rates, mortality rates, and case fatality rates by both home and incident address.

Statistical analysis for this study was performed using SPSS 22 (IBM, Armonk, NY). Parametric data is presented as mean ± standard deviation (SD). We used student *t*-test and Bonferroni tests for multiple comparisons to identify differences by race in age, sex, admission, multiple GSW, mortality, and ICU length of stay for patients. We fitted ordinary least squares (OLS) regression models to identify trends in firearm violence by census tract during the time period. Stepwise regression was used as an exploratory tool to guide the introduction of covariates in our modeling approach. We also used GeoDa 1.8.2 (Arizona State University, Tempe, AZ) to improve model specification by fitting spatial regression models, which introduce a spatial lag term to control for spatial effects. Two-tailed statistical significance was set at α = .05. The Institutional Review Board at the University of Miami approved this study.

## Results

**Fig. 1:**
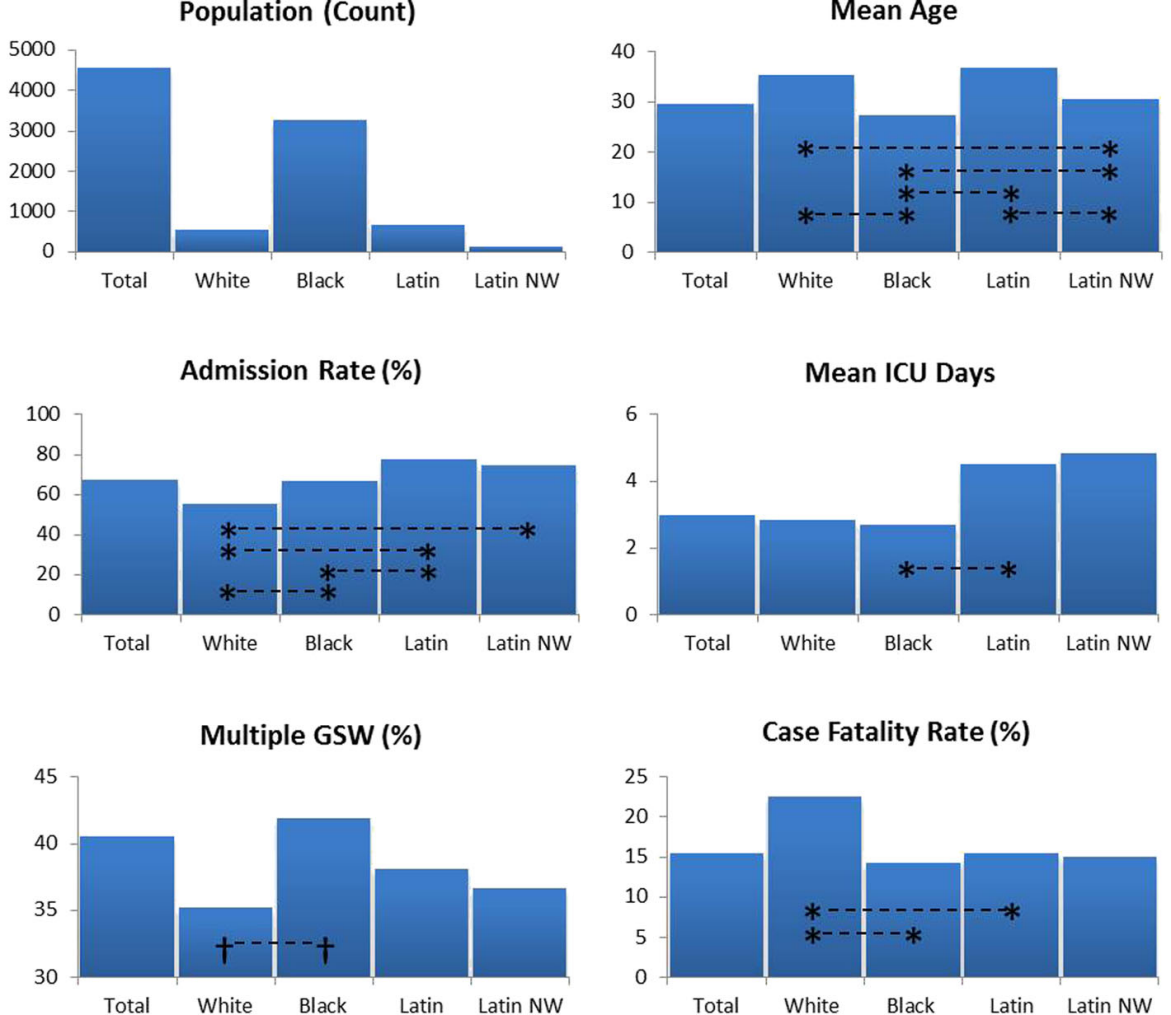
Patient characteristics by race/ethnicity (Latin NW = Latin non-white) with statistically significant pairs from Bonferroni test of multiple comparisons († *P* < .05, * *P* < .01)

Descriptive characteristics of the study population are summarized in Fig. 1. The cohort was 91.2% male with an average age of 29.51 ± 12.6. The distribution of race was 71.8% black, 14.1% Latin, 11.8% white, and 2.2% Latin non-white. This cohort differs significantly from the MDC population that is 19% black (*P <* 0.001). Injury location included abdomen (19.3%), chest (11.3%), head (5.5%), back (5%), pelvis (5.4%), lower extremities (15.9%), upper extremities (3.3%), and 40.5% of patients experienced multiple GSWs. Average ICU length of stay was 2.97 ± 11.2 days. Sixty-seven percent of all GSW patients were admitted to the hospital, and the case fatality rate was 15.4%.

GSW patient characteristics were stratified by patient race (Fig. 1). There was no significant difference in sex between all races. Average age by race was: white (35.2 ± 15.2), black (27.2 ± 10.8), Latin (36.6 ± 14.1), and Latin non-white (30.98 ± 13.2). There was a significant difference in age between black patients and white (*P* < 0.001), Latin (*P* < 0.001), and Latin non-white (*P* < 0.001) patients. The differences in means indicated that on average black patients present 8 years younger than white patients, 9.43 years younger than Latin patients, and 3.80 years younger than Latin non-white patients.

There were significant differences in clinical outcomes between patients of different race. Admission to the trauma center varied by race: 55.2% white, 66.3% black, 77.3% Latin and 74.3% for Latin non-white, with a significant difference of white patients being admitted less frequently than black (*P* < 0.001), Latin (*P* < 0.001), and Latin non-white (*P* = 0.001), with Latin patients also more likely to be admitted than black patients (*P* < 0.001). There was a significant difference in length of ICU stay between black (2.66 ± 9.92) and Latin (4.47 ± 16.4), with an average of 1.8 days difference (*P* < 0.001). White patients had a higher case fatality rate of 22.5% compared to black (14.2%, *P* < 0.001) and Latin (15.4%, *P* = 0.004) patients. Almost 42% of black patients sustained multiple GSWs, a significantly higher frequency than for white patients (35.2%, *P* = 0.019).

**Fig. 2:**
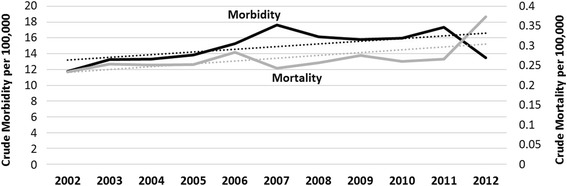
Annual crude morbidity and mortality attributable to GSW in MDC per 100,000 persons. Dotted lines indicate corresponding trend lines

**Fig. 3:**
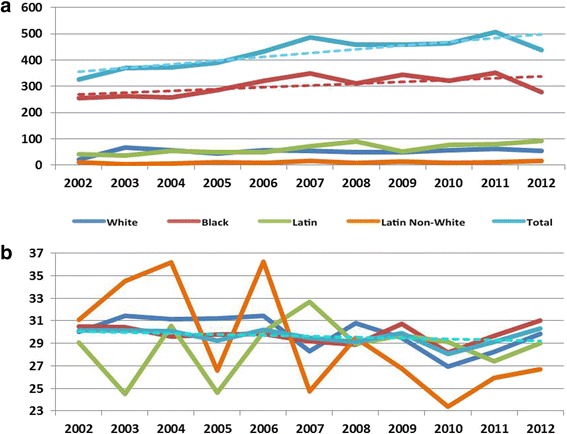
Annual trends in frequency of (**a**) injury, and (**b**) age, by race. Dotted lines indicate corresponding trend lines

The crude morbidity rate of GSW injury over the 11-year period was 15 per 100,000 persons with a crude mortality rate of 0.27 per 100,000 persons. Both morbidity and mortality increased modestly over the study period (Fig. 2). We also observed several demographic trends in firearm violence as shown in Fig. 3. Overall, an increase in firearm-related injury was observed from 2002 to 2012. However, this trend was not evident within all racial groups. There was a clear increase in the black population, and a moderate increase in Latin populations. Trends in white and Latin non-white remained the same over the 11-year period. There was a slight decrease in overall age, however Latin and Latin non-white populations varied greatly by year (*P* = 0.027).

**Fig. 4:**
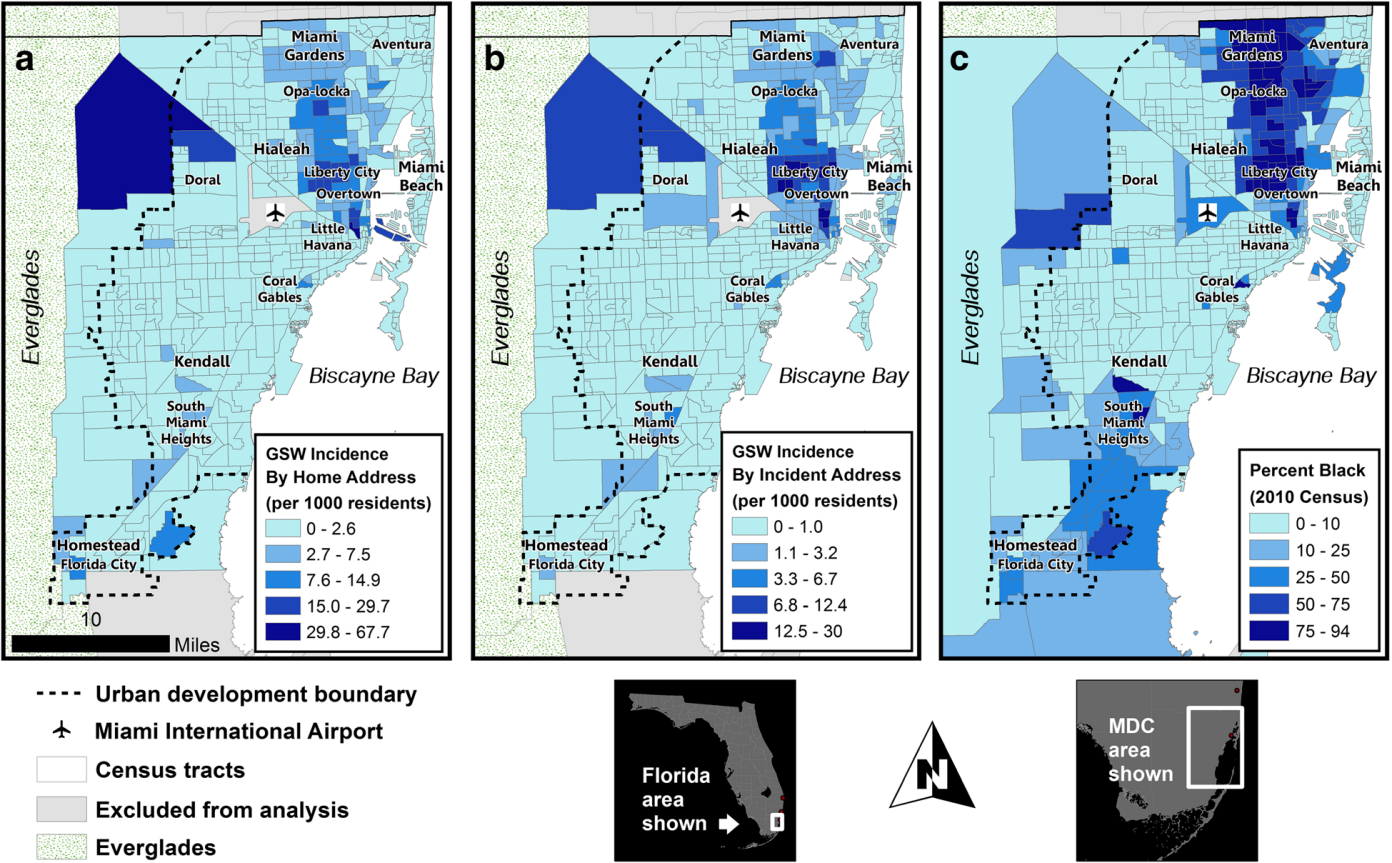
GSW incidence by (**a**) home address and (**b**) incident address shows a majority of events occurring in northeast Miami-Dade County (MDC) coinciding with the distribution of (**c**) percent black population

Of the 4,546 patients that presented to the trauma center, 3,908 (86.0%) were successfully geocoded using the home address and 2,788 (61.3%) were geocoded using incident address. Four census tracts were eliminated from the analysis due to low populations with a nominal number of events that would otherwise skew rates of injury. The remaining 514 census tracts were used for analysis. Figure 4 reveals a concentration of GSW incidence by home and incident address in black neighborhoods such as Opa-Locka, Liberty City, and Overtown.

**Fig. 5:**
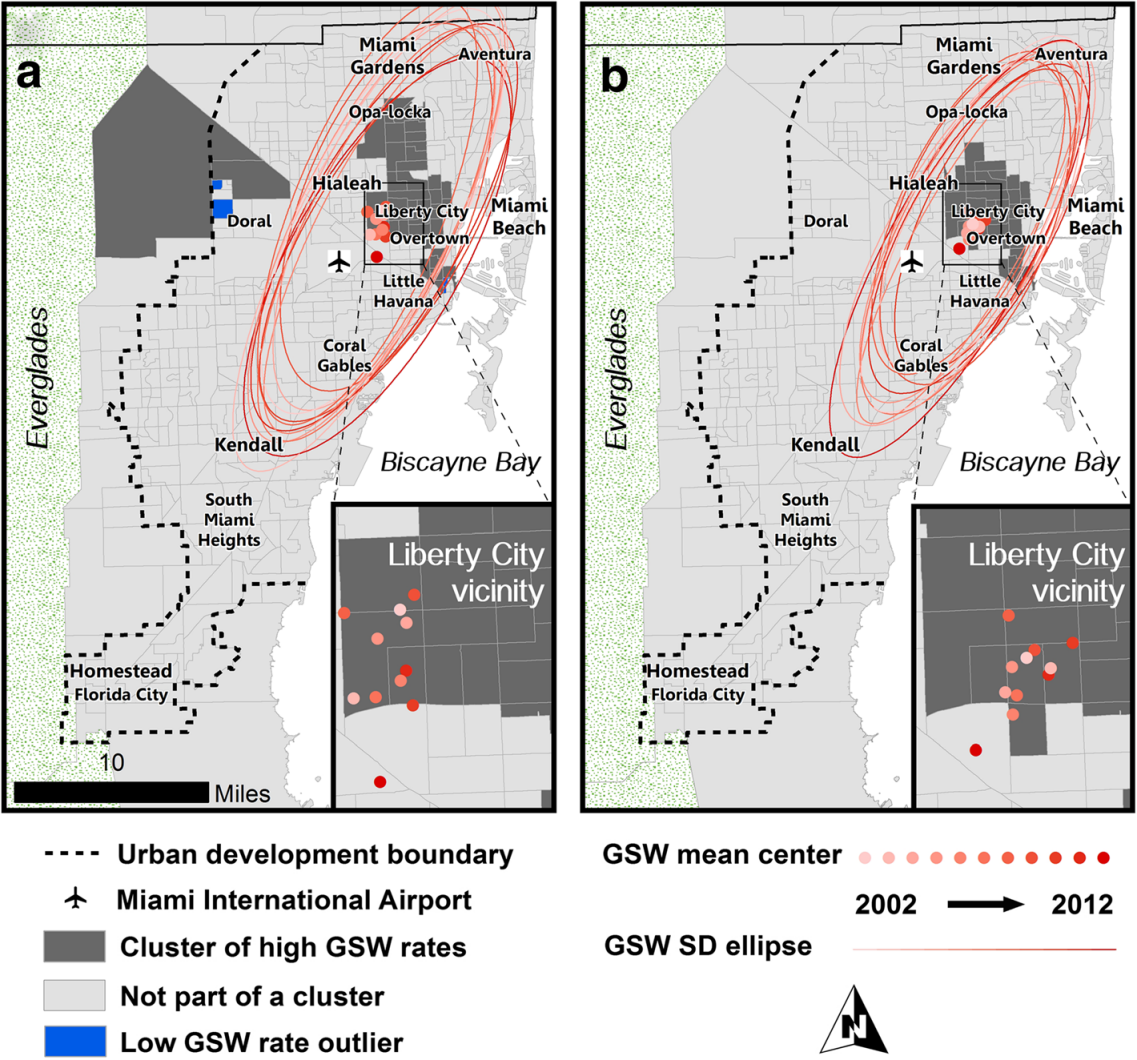
Spatial clusters and descriptive spatial statistics of high GSW rates by (**a**) home address and (**b**) incident address. Mean centers and standard deviation ellipses highlight the persistent epicenter of violence in the Liberty City vicinity over the 11 year study period

We calculated the mean center of all GSW incidents and standard distance ellipses of their spatial distributions by year using the locations of the home and incident addresses. The mean center represents the average location of all GSW incidents for each year, and the standard distance ellipse represents the compactness of the distribution as an ellipse containing 68% of the incidents (those within 1 standard deviation of the *x* and *y* coordinates of the mean center). The absence of any temporal trends in the geographic distribution of both home and incident locations is presented in Fig. 5. The mean center and size and orientation of the standard ellipses are geographically persistent throughout the study period, with the mean center rotating through five contiguous census tracts in the Liberty City vicinity.

The Moran’s I statistic was used to assess global patterns of spatial autocorrelation. Our analysis revealed statistically significant global clustering of frequency rates using home address (*I* = 0.3981, *Z* = 17.0256, *P* < 0.001), and for incident address (*I* = 0.61764, *Z* = 25.78094, *P* < 0.001). Mortality rates were significantly clustered by census tract for both home and incident address, respectively (*I* = 0.0644, *Z* = 3.6855, *P* < .001; *I* = 0.6207, *Z* = 58.1321, *P* < .001), although there was no pattern for case fatality rates (*I* = 0.030593, *Z* = 1.324432, *P* = 0.18536 for home address; *I* = −0.0011, *Z* = 0.03452, *P* = 0.9725 for incident address). The spatial statistic LISA was then used to identify the specific locations of spatial clusters and potential outliers for incidence, mortality, and case fatality rates. Maps of statistically significant spatial clusters and outliers identified by the LISA statistic are presented in Fig. 5. These maps indicate high clustering for incidence rates by home and incidence address. Figure 5 depicts all incidents including fatalities; a separate spatial cluster analysis of just mortality revealed virtually identical clusters that were slightly more concentrated. Outliers in both home address incidents in western census tracts near Hialeah; these tracts are mostly industrial and therefore appear as outliers due to very low numbers of events and a small baseline population. The distance between home address and incident address was calculated for a subset of patients for whom both addresses were available. On average, patients that were shot within the incident cluster lived within 1.36 miles, while those whose incident occurred outside of the cluster lived 3.35 miles from their incident location.

We conducted correlation and multivariate regression analyses to examine the relationship between incidence rates in home address census tracts and various potential predictors. Three census tracts containing airports or industrial areas with a population was fewer than 10 residents were removed from analysis, resulting in a final sample size of 510 census tracts. The GSW incidence rate was positively and significantly correlated with percent black (*r* = 0.550, *P* < 0.001), percent single family (*r* = 0.496, *P* < 0.001), and percent unemployment (r = 0.356, *P* < 0.001) and negatively and significantly correlated with average income (r = −0.306, *P* < 0.001), percent Hispanic (*r* = −0.354, *P* < 0.001) and median age (*r* = −0.187, *P* < 0.001). Multivariate regression diagnostics indicated borderline multicollinearity for both percent black and percent Hispanic, respectively (variance inflation factor [VIF] = 5.123, VIF = 3.114), thus percent Hispanic was removed as a predictor. An ordinary least squares regression model of census tract level GSW incidence on black population, the percentage of single-parent households, and median age accounted for approximately 36% in the variation of GSW incidence (*R* ^2^ = 0.36, *F* = 94.31, Std Error = 4.47; see Table 1) and was the most parsimonious model. Census tracts with a higher percent of black population (*β* = 0.078, *t* = 7.16, *P* < 0.001), percent of single families (*β* = 0.197, *t* = 6.56, *P* < 0.001), and median age (*β* = 0.170, *t* = 3.92, *P* < 0.001), were more likely to have higher incidence rates. However, spatial analysis of model residuals using Moran’s *I* revealed positive spatial autocorrelation (*I* = 0.265, *Z* = 11.64, *P* < 0.001), meaning that spatial dependence limited proper specification of the OLS model. We proceeded to fit a spatial lag model using a spatial weights matrix with neighbors defined by first-order contiguity among census tracts (see Table 2). Again, census tracts with a higher percent of black population (*β* = 0.055, *Z* = 4.87, *P* < 0.001), percent of single families (*β* = 0.177, *Z* = 6.11, *P* < 0.001), and median age (*β* = 0.179, *Z* = 4.32, *P* < 0.001), were more likely to have higher incidence rates. The model *R* ^2^ increased from .36 to .42 but still failed the Breusch-Pagan test for heteroscedasticity, which suggests additional unresolved spatial dependence.

**Table 1:** Ordinary least squares regression model showing census tract-level predictors of GSW incidence by home address (*n* = 510 tracts)

Characteristic	*β* (SE)	*t*	*P*-value	95% CI for *β*
Constant	−9.81 (2.02)	−4.86	<.001	−13.77, −5.84
Black population (%)	.08 (.01)	7.16	<.001	.06, .10
Single parent households (%)	.20 (.03)	6.56	<.001	.14, .26
Median age (years)	.17 (.04)	3.92	<.001	.09, .26
Model diagnostics: *R* ^2^ = .36

**Table 2:** Spatial lag regression model showing census tract-level predictors of GSW incidence by home address (*n* = 510 tracts)

Characteristic	*β* (SE)	*Z*-score	*P*-value
Constant	−10.17 (1.93)	−5.27	<.001
Spatial Lag Term (Rho)	.35 (.06)	6.16	<.001
Black population (%)	.06 (.01)	4.87	<.001
Single parent households (%)	.18 (.03)	6.11	<.001
Median age (years)	.18 (.04)	4.32	<.001
Model diagnostics: AIC = 2937.61, *R* ^2^ = .42			

## Discussion

This analysis sought to identify the socio-demographic, spatial, and temporal trends that occur in firearm-related injury within MDC. Our findings suggest that there are clear racial, economic, and geographic disparities in firearm violence. Our study population was disproportionately comprised of young, black males relative to the MDC population. Black patients presented at the trauma center at a much younger average age than white and Latin patients and were more likely to present with multiple GSWs compared to white patients. Firearm violence within the black population steadily increased over the course of the eleven-year study period, which drove the county-wide trend, while the average age of all patients decreased. Geospatial analysis indicated that while the number of incidents is increasing, there was virtually no change in the geographic distribution of firearm injury over the study period. Firearm violence and the neighborhoods where individuals both reside and participate in violence persisted in a handful of census tracts in MDC over the study period. These census tracts were clustered in predominately low-income, black neighborhoods in Opa-Locka, Liberty City, and Overtown in the northeast region of MDC. In addition, crude mortality (not presented) was also spatially clustered in these specific areas. Regression analyses indicated that the incidence rate at the tract level was significantly associated with a higher percentage of the black resident population, higher median age, and higher percentage of single-family homes.

These findings corroborate recent studies that demonstrate clear racial disparities in firearm-violence throughout large metropolitan areas in the United States, specifically in young, black males [7]. The results also concur with national trends in urban–rural and racial-ethnic disparities over the same study period [1]. While the GSW incidence rate is below the national average across most of MDC, several intense pockets of GSW injury in MDC have morbidity rates 5–10 times higher than the national age-adjusted average rate. The disparities between racial groups are possibly related to social disorganization theory that describes a variety of social pressures. This theory suggests that crime is likely a function of neighborhood dynamics that are favorable to crime, and not necessarily a function of the individuals within the neighborhoods. Black communities are theorized to have higher rates of firearm-violence because they are subjected to situations that are conducive to increased violence including poverty, or a lack of employment opportunities and social capital [7, 30–35]. Peer pressure plays an important role in the incidence of gun violence by allowing the cultural transmission of delinquent values. Research indicates that black, urban neighborhoods are more likely to cultivate social pressures to gain peer respect by appearing dominant and “bad” in social interactions by carrying a gun [7, 35–37]. This phenomenon, referred to as the “code of the street,” may increase the risk for firearm injury in black youth compared to other racial groups [38]. In addition, recent studies of violence in MDC indicate that while white males are more likely to carry a weapon, their weapon of choice is typically a knife, whereas guns are more commonly carried by black males [39]. Because most firearm violence is intra-racial, black residents are more likely to face assailants armed with guns [7, 40]. It is this “code of the street” embedded in black urban youth which— along with disproportionate and increasing poverty in these communities—may explain not only the rising incidence of gun violence, but also the increased likelihood of sustaining multiple GSWs. It is possible that multiple gunshots are an attempt by the assailant to appear resilient and merciless among peers, but this is beyond the scope of this study and requires further research.

Within this analysis, white patients experienced worse case fatality rates. These findings are not consistent with other studies that have described higher mortality rates in firearm-related injury among black patients [9–12]. We do not currently know why this disparity exists among MDC’s white population relative to other cities, but event, assailant, and victim characteristics may contribute to this difference, as may the white population’s minority status in MDC, or bias in reporting. Further research in MDC is required to identify possible explanations for this difference.

Spatial cluster analysis revealed distinct clusters of GSW incidence rate by home address and incident address, as well as mortality rates (not presented). This suggests that not only are events of violence clustered in predominantly black neighborhoods in northeast MDC, but also that patients residing in or participating in violence within these neighborhoods are more likely to die from these violent events. Descriptive spatial statistics (mean location and distribution) of GSWs by year revealed that despite slight shifts in socio-demographics during the eleven-year period, the epicenter of gun violence was quite focal, persisting in just a few census tracts in MDC. This concentration of gun violence suggests the possibility of a strong neighborhood effect in MDC, which is a clear topic for future investigation. Patients also tended to participate in violence within a few miles of their home, indicating that violence in Miami is typically maintained within specific neighborhoods. This is consistent with previous studies that have shown clustering in predominately low-income, black neighborhoods and a high degree of stability of crime at micro scales over time despite demographic change [41, 42]. It is important to recognize that the increase in firearm injury over the eleven-year study period mirrors poverty and inequality rates over time, which disproportionately affects black communities and differential access to economic resources that perpetuate violence. This likely relates to research on social disorganization theory that suggests that crime occurs in particular areas of the city due to physical attributes of an area, among other factors such as limited economic options. These disparities within MDC are driven by a history of urban planning and divestment that caused the creation of black, urban ghettos and disenfranchised communities in the neighborhoods of Liberty City and Overtown, where the spatial clusters of GSWs were centered. While these analyses used census tracts as an operational scale for gun violence, this study found that certainly not every block within a high-GSW census tract experienced high levels of violence, while others had none. This is largely related to physical and social characteristics of particular blocks within a neighborhood [22]. Further sub-analysis of this data in areas with high GSW rates will indicate specific blocks that would benefit from targeted prevention programs that aim to improve urban spaces and diversify economic options.

The most alarming findings of this study are (1) the spatial regression analysis that indicates that the percentage of black residents, percentage of single family households, and median age accounted for 42% of the variation in GSW incidence rate by home address, and (2) the focal clustering of gun violence over the study period. As previously mentioned, race and neighborhood are interconnected within MDC, and this racial segregation is due to a history of racially-charged policies that have exacerbated violence. The persistent clustering of firearm-injury over the study period shows an alarming lack of community and political engagement and gun control policies that might normally contribute to some geographical variation in gun violence patterns, and ultimately a reduction in mortality. Such inaction in the face of well-documented need arguably perpetuates a history of institutionalized racism in Miami. Our findings thus represent a call for urgent intervention that must address key risk factors in a very small area of MDC. Such interventions could have a significant public health impact on interrupting this epidemic of gun violence and serve as a model for other cities [43]. Gun violence interventions would provide tangible benefits to Miami as a whole through better public safety, subsequent economic opportunities, and improved social equity between minority groups. Targeted public health interventions by municipal— and state-level policy makers, starting with employment opportunities, economic inclusion, poverty reduction programs, and efforts to reduce structural racism, would likely have a significant county-wide public health impact.

There are several limitations to this analysis. First, this data does not include the assailant and thus does not accurately represent the nature of the crime. Furthermore this does not indicate if these injuries are related to gang and drug violence or if they are a result of domestic violence. This information could be used to investigate possible differences in crime nature and interpersonal aggression within and between racial groups. In addition, this data set did not include income classification, wealth and occupation, which may provide further clarity on firearm trends in MDC. Occupation was included in the original data set, however these data were missing for 86.6% of patients and therefore unreliable. Census tract-level income data was modeled in this study, but it was not significantly associated with GSW incidence because not every low-income neighborhood experiences gun violence; individual-level wealth would provide a more robust indicator of economic access. Finally, MDC constitutes unique ethnic enclaves and a diverse population that includes many biracial individuals. While our data set did include a small percentage of Latin non-white, this data set does not include differences among ethnic groups within these racial categories, and thus there is tremendous subjectivity in the classification of race. Further research addressing individual-level socio-demographic indicators such as occupation, income, and ethnicity could provide a clearer picture of GSW risk factors.

## Conclusion

Our study is the first of its kind to observe firearm-related injury from a patient perspective at a Level I Trauma Center in MDC. There is a high prevalence of firearm-related injury among young, black males that may be explained by neighborhood social structures and employment. The geo-demographic distribution of firearm-related injury has not changed in at least 11 years with substantial geographical overlap between incident location and patient address. In contrast, white patients had worse clinical outcomes including greater mortality, although there is currently no evidence to support an explanation; this will require further investigation. But these findings do provide a new evidence base for public policies that address the patterns of firearm violence within MDC. With a case fatality rate of 15.3% at this Level I Trauma Center, preventative measures are necessary and overdue. As recent firearm violence and its ties to racial perceptions continues to flood our national media, a deeper understanding of socio-demographic and economic characteristics can help promote political change and identify modifiable risk factors for reducing violence within local communities.

## Abbreviations

GIS: Geographic information system
GSW: Gunshot wound
ICU: Intensive care unit
IDC: International classification of disease
LISA: Local indicators of spatial autocorrelation
MDC: Miami-dade county
OLS: Ordinary least squares
SD: Standard deviation
